# Recent Developments in NAFLD

**DOI:** 10.3390/ijms23052882

**Published:** 2022-03-07

**Authors:** Alessandro Mantovani, Andrea Dalbeni

**Affiliations:** 1 Section of Endocrinology, Diabetes and Metabolism, Department of Medicine, Azienda Ospedaliera Universitaria Integrata Verona, Piazzale Stefani, 1, 37126 Verona, Italy; 2 Section of General Medicine C and Liver Unit, University of Verona, 37134 Verona, Italy; andrea.dalbeni@aovr.veneto.it

The aim of our Special Edition, entitled “*Nonalcoholic Fatty Liver Disease/Metabolic Associated Fatty Liver Disease: New Insights*”, is to point out recent developments in the area of NAFLD pathogenesis and treatment. In this regard, it is important to remember that NAFLD is, to date, the most frequent chronic liver disease observed in clinical practice worldwide [1,2]. It is estimated that NAFLD affects roughly 25–30% of adults in the general population [3] and nearly 70% of patients with type 2 diabetes (T2DM) [4,5]. Of note, in recent decades it has become clear that NAFLD is associated with liver-related complications, but also with metabolic, cardiovascular and renal complications [2]. In this setting, the most common cause of death in NAFLD patients is cardiovascular disease [6]. Although the pathogenesis of NAFLD is quite complicated and not completely understood yet, it is established that NAFLD is closely linked to insulin resistance, obesity and T2DM [2]. For this reason, in 2020, several authors and some scientific societies proposed the change in the terminology from NAFLD to metabolic-associated fatty liver disease (MAFLD), as well as an update of the definition of this fatty liver disease [7].

Table 1 summarizes the articles published in our Special Edition. Regarding the NAFLD pathogenesis, experimentally, Nakade et al. have demonstrated that central corticotropin-releasing factor (CRF) can affect hepatic de novo lipogenesis and inflammation-related gene expression by the sympathetic–noradrenergic nervous system in rats [8]. Given that de novo lipogenesis and inflammation are relevant mechanisms involved in the pathogenesis of NAFLD and its advanced forms [1,5], this evidence further expands our knowledge, suggesting that the brain–liver axis may, at least in part, modulate the development and progression of hepatic steatosis. In another work of this Special Edition, Nakade et al. reported that the sympathetic nervous system may further modulate hepatic lipid metabolism by regulating adrenergic receptor activation [9]. Specifically, alpha-adrenergic receptors may play an inhibitory role in hepatic steatosis in mice with NAFLD [9]. In addition, experimentally, Kim et al. also reported that Jumonji domain-containing protein 2B (JMJD2B) may modulate LXRα-mediated lipogenesis by various mechanisms, contributing to hepatic steatosis [10].

Among different mechanisms implicated in the NAFLD pathogenesis, hepatic senescence also seems to play a role [11]. In recent years, several senescence processes were found, including replicative and stress-induced senescence [12]. In non-aged mice models, Moustakas et al. highlighted the importance of stress-induced senescence in the development of hepatic steatosis [13]. In that study, they found that liver fat accumulation and increased hepatic mRNA expression of steatosis-related genes may be occurred with hepatic stress-induced senescence [13]. This evidence might be useful for future therapy for NAFLD patients.

Environmental factors can also play a role in the development and progression of NAFLD by various mechanisms [1]. In a recent review, Negi et al. focused on the potential role of flame retardants (FRs), which are anthropogenic chemicals or mixtures used to inhibit the spread of fire, in the development and progression of NAFLD by modulating immune and inflammatory signaling [14].

As mentioned above, NAFLD is now considered a multi-systemic disease [2]. In a narrative review, Hatasa et al. summarized the relationship between NAFLD and periodontal disease, highlighting that oral health can be also important for systemic health in patients with NAFLD [15].

Regarding NAFLD treatment, Wilson et al. showed that in obese mice with NAFLD, two-week, isocaloric, time-restricted feeding may decrease liver inflammation, without significant weight loss [16]. In this regard, it is important to remember that time-restricted feeding (TRF) procedures, such as intermittent fasting, have obtained scientific interest as potential treatments for obesity and obesity-related diseases. Given that, to date, information regarding the benefits of time-restricted feeding in NAFLD is still scarce, the study by Wilson et al. provides novel and important data on this topic. Finally, we have summarized the recent developments in the area of NAFLD treatment. In our narrative review, we have focused on evidence regarding the use of glucose-lowering drugs (i.e., pioglitazone, glucagon-like peptide-1 (GLP-1) receptor agonists, sodium–glucose co-transporter-2 (SGLT-2) inhibitors), antioxidants, statins, bile and non-bile acid farnesoid X activated receptor (FXR) agonists for the treatment of NAFLD [17]. However, in the light of multiple pathways involved in the NAFLD pathogenesis [5], the combination of different therapies might be useful for treating NAFLD patients [18]. In this setting, as suggested by several authors [5,18,19], we believe that a holistic approach in managing NAFLD is now pivotal.

## Figures and Tables

**Table 1:** Articles published in the Special Edition entitled “*Nonalcoholic Fatty Liver Disease/Metabolic Associated Fatty Liver Disease: New Insights*”.

Authors	DOI	Year	Article Type	Title	Main Results
Nakade et al.	10.3390/ijms22083940	2021	Article	*Effect of Central Corticotropin-Releasing Factor on Hepatic Lipid Metabolism and Inflammation-Related Gene Expression in Rats*	In rats, corticotropin-releasing factor affects hepatic de novo lipogenesis and inflammation-related gene expression via the sympathetic–noradrenergic nervous system
Nakade et al.	10.3390/ijms21249392	2020	Article	*Effect of Adrenergic Agonists on High-Fat Diet-Induced Hepatic Steatosis in Mice*	Phenylephrine inhibits hepatic steatosis via the stimulation of β-oxidation and autophagy in the liver
Kim et al.	10.3390/ijms21218313	2020	Article	*Histone H3K9 Demethylase JMJD2B Plays a Role in LXRα-Dependent Lipogenesis*	Jumonji domain-containing protein 2B (JMJD2B) may modulate LXRα-mediated lipogenesis by various mechanisms, contributing to hepatic steatosis
Moustakas et al.	10.3390/ijms22073446	2021	Article	*Hepatic Senescence Accompanies the Development of NAFLD in Non-Aged Mice Independently of Obesity*	In mice, senescence (including stress-induced senescence) may play a role in the NAFLD development
Wilson et al.	10.3390/ijms21239156	2020	Article	*Two-Week Isocaloric Time-Restricted Feeding Decreases Liver Inflammation without Significant Weight Loss in Obese Mice with Non-Alcoholic Fatty Liver Disease*	Two-week, isocaloric time-restricted feeding decreased liver inflammation, without significant weight loss or reductions in hepatic steatosis, in obese mice with NAFLD
Negi et al.	10.3390/ijms22084282	2020	Review	*Flame Retardants-Mediated Interferon Signaling in the Pathogenesis of Nonalcoholic Fatty Liver Disease*	Flame retardants (i.e., anthropogenic chemicals or mixtures used to inhibit the spread of fire) may play a role in the development of NAFLD by modulating immune and inflammatory signaling
Hatasa et al.	10.3390/ijms22073728	2021	Review	*Relationship between NAFLD and Periodontal Disease from the View of Clinical and Basic Research, and Immunological Response*	Oral health is essential in patients with NAFLD
Mantovani et al.	10.3390/ijms22052350	2021	Review	*Treatments for NAFLD: State of the art*	This narrative review discusses the different available approaches with the potential to prevent and treat NAFLD and its advanced forms

## References

[1] Powell E.E., Wong V.W., Rinella M. (2021). Non-alcoholic fatty liver disease. Lancet.

[2] Mantovani A., Scorletti E., Mosca A., Alisi A., Byrne C.D., Targher G. (2020). Complications, morbidity and mortality of nonalcoholic fatty liver disease. Metabolism.

[3] Le M.H., Yeo Y.H., Li X., Li J., Zou B., Wu Y., Ye Q., Huang D.Q., Zhao C., Zhang J. 2019 global NAFLD prevalence—A systematic review and meta-analysis. Clin. Gastroenterol. Hepatol..

[4] Younossi Z.M., Golabi P., de Avila L., Paik J.M., Srishord M., Fukui N., Qiu Y., Burns L., Afendy A., Nader F. (2019). The global epidemiology of NAFLD and NASH in patients with type 2 diabetes: A systematic review and meta-analysis. J. Hepatol..

[5] Stefan N., Cusi K. A global view of the interplay between non-alcoholic fatty liver disease and diabetes. Lancet Diabetes Endocrinol.

[6] Mantovani A., Csermely A., Petracca G., Beatrice G., Corey K.E., Simon T.G., Byrne C.D., Targher G. (2021). Non-alcoholic fatty liver disease and risk of fatal and non-fatal cardiovascular events: An updated systematic review and meta-analysis. Lancet Gastroenterol. Hepatol..

[7] Mantovani A., Dalbeni A. (2020). NAFLD, MAFLD and DAFLD. Dig. Liver Dis..

[8] Nakade Y., Kitano R., Yamauchi T., Kimoto S., Sakamoto K., Inoue T., Kobayashi Y., Ohashi T., Sumida Y., Ito K. (2021). Effect of Central Corticotropin-Releasing Factor on Hepatic Lipid Metabolism and Inflammation-Related Gene Expression in Rats. Int. J. Mol. Sci..

[9] Nakade Y., Kitano R., Yamauchi T., Kimoto S., Sakamoto K., Inoue T., Kobayashi Y., Ohashi T., Sumida Y., Ito K. (2020). Effect of Adrenergic Agonists on High-Fat Diet-Induced Hepatic Steatosis in Mice. Int. J. Mol. Sci.

[10] Kim J.H., Jung D.Y., Kim H.R., Jung M.H. (2020). Histone H3K9 Demethylase JMJD2B Plays a Role in LXRalpha-Dependent Lipogenesis. Int. J. Mol. Sci..

[11] Ogrodnik M., Miwa S., Tchkonia T., Tiniakos D., Wilson C.L., Lahat A., Day C.P., Burt A., Palmer A., Anstee Q.M. (2017). Cellular senescence drives age-dependent hepatic steatosis. Nat. Commun..

[12] Gorgoulis V., Adams P.D., Alimonti A., Bennett D.C., Bischof O., Bishop C., Campisi J., Collado M., Evangelou K., Ferbeyre G. (2019). Cellular Senescence: Defining a Path Forward. Cell.

[13] Moustakas I.I., Katsarou A., Legaki A.I., Pyrina I., Ntostoglou K., Papatheodoridi A.M., Gercken B., Pateras I.S., Gorgoulis V.G., Koutsilieris M. (2021). Hepatic Senescence Accompanies the Development of NAFLD in Non-Aged Mice Independently of Obesity. Int. J. Mol. Sci..

[14] Negi C.K., Khan S., Dirven H., Bajard L., Blaha L. (2021). Flame Retardants-Mediated Interferon Signaling in the Pathogenesis of Nonalcoholic Fatty Liver Disease. Int. J. Mol. Sci..

[15] Hatasa M., Yoshida S., Takahashi H., Tanaka K., Kubotsu Y., Ohsugi Y., Katagiri T., Iwata T., Katagiri S. (2021). Relationship between NAFLD and Periodontal Disease from the View of Clinical and Basic Research, and Immunological Response. Int. J. Mol. Sci..

[16] Wilson R.B., Zhang R., Chen Y.J., Peters K.M., Sawyez C.G., Sutherland B.G., Patel K., Kennelly J.P., Leonard K.A., Jacobs R.L. (2020). Two-Week Isocaloric Time-Restricted Feeding Decreases Liver Inflammation without Significant Weight Loss in Obese Mice with Non-Alcoholic Fatty Liver Disease. Int. J. Mol. Sci..

[17] Mantovani A., Dalbeni A. (2021). Treatments for NAFLD: State of Art. Int. J. Mol. Sci..

[18] Dufour J.F., Caussy C., Loomba R. (2020). Combination therapy for non-alcoholic steatohepatitis: Rationale, opportunities and challenges. Gut.

[19] Byrne C.D., Targher G. (2021). Non-alcoholic fatty liver disease is a risk factor for cardiovascular and cardiac diseases: Further evidence that a holistic approach to treatment is needed. Gut.

